# Lessons for the clinical nephrologist: unilateral renal artery stenosis presenting with hyponatremic hypertensive syndrome and posterior reversible encephalopathy syndrome in a child

**DOI:** 10.1007/s40620-025-02320-7

**Published:** 2025-06-10

**Authors:** Muhammad Adel Sayed, Mohamed Ezzat Al Ghwass, Ashraf Sayed Kamel, Remon Magdy Yousef Awad

**Affiliations:** Faculty of Medicine, Fayoum University, Fayoum City, Egypt

**Keywords:** Hyponatremia, Renovascular hypertension, Renal artery stenosis, Posterior reversible encephalopathy syndrome

## The case

A 5-year-old boy with no significant medical history presented after four generalized tonic–clonic seizures without fever one day prior to admission. He had a one-month history of headache and a 2-year history of polyuria, polydipsia, and poor weight gain. On examination, his weight and height were both at the 3rd percentile (15 kg, 99 cm). He appeared mildly dehydrated and had markedly elevated blood pressure (BP) (205/150 mmHg). Neurological examination was unremarkable.

**Fig. 1:**
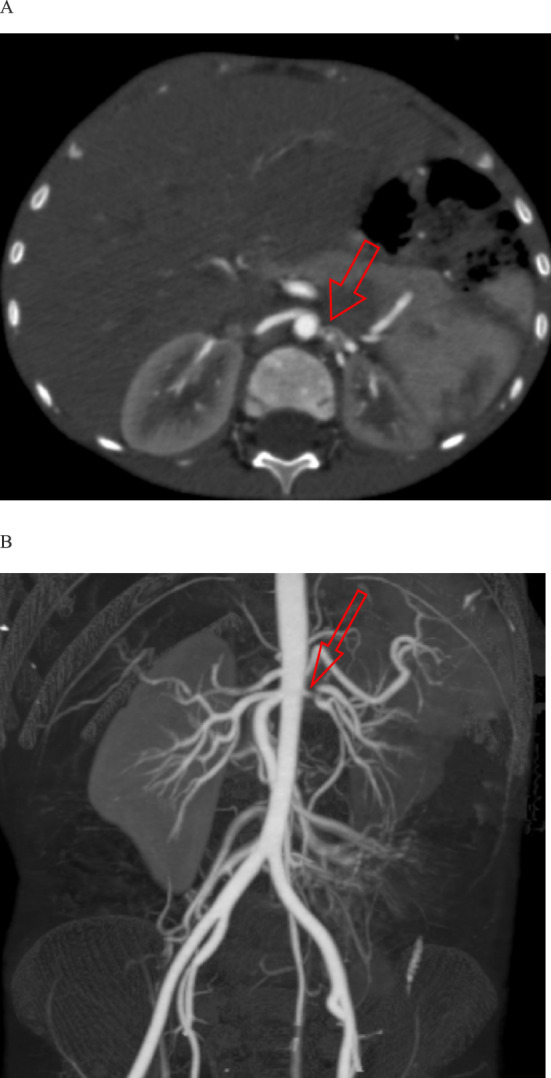
CT angiography (images **A**, **B**) showing left renal artery stenosis with small left kidney and enlargement of the right kidney

Initial laboratory tests (Table 1) showed hyponatremia, hypokalemia, and metabolic alkalosis. Urinary Na was 48 mEq/L, and microalbuminuria (urine albumin/creatinine ratio of 277 mg/g) was present. Brain magnetic resonance imaging (MRI) revealed a left small area of parieto-occipital demyelination consistent with posterior reversible encephalopathy syndrome (Fig. 1S, supplemental material). Further workup showed markedly elevated plasma renin (128 pg/mL; normal: 3.5–36) and aldosterone (471 pg/mL; normal: 14.2–156). Complement levels (C3, C4) were normal, ANCA was negative, and ANA was weakly positive. Fundus examination showed hypertensive retinopathy and papilledema in the left eye only. Echocardiography revealed left ventricular hypertrophy.

**Table 1:** Laboratory tests

Serum parameters	Day 0	Day 1	Day 2	Day 4	Day 5	Day 6	Day 8
Urea (15–40 mg/dL)	26	28	26				
Creatinine (0.3–0.7 mg/dL)	0.6	0.7	0.4				
Na (135–145 mEq/L)	124	120	122	121	128	130	131
K (3.6–5.2 mEq/L)	2.6	2.4	3	3.8	4.4	3.6	4
Serum osmolarity (285–295 mOsmol/kg)	258						
Ca (8.8–10.8 mg/dL)	8.8						
pH (7.35–7.45)	7.7	7.6	7.6			7.57	7.58
pCO2 (35–45 mmHg)	20	27	19			28	29
HCO3 (22–26 mEq/L)	30	26	26			28	29
Plasma renin (3.5–36 pg/mL)				More than 128			
Aldosterone (14–156 pg/mL)				471			
C3 (80–178 mg/dL)				129.7			
C4 (12–42 mg/dL)				34.1			
ANA				Weak positive (1/40)			
c-ANCA, p-ANCA				Negative			
Urinary chloride (normal: below 20 mEq/L)		52					
Urinary Na (normal: below 20 mEq/L)		48					
Urinary K (normal: below 10 mEq/L)		30					

*ANA* Antinuclear antibody, *c-ANCA* cytoplasmic anti-neutrophil cytoplasmic antibody, *p-ANCA* perinuclear anti-neutrophil cytoplasmic antibody, *C3, C4* complement components 3 and 4

During admission, he had persistent polyuria (10 ml/kg/hr initially, decreasing to 4–5 ml/kg/hr). Abdominal ultrasounds revealed a hyperechoic right kidney (8.5 cm in length) and a smaller left kidney (7 cm in length). Doppler ultrasounds suggested left renal artery stenosis. The co-existence of hyperreninemic hypertension, hyponatremia, hypokalemia and natriuresis is highly suggestive of hyponatremic hypertensive syndrome. Computed tomography (CT) angiography confirmed a short, proximal stenotic segment (~ 6 mm) in the left renal artery, causing 60–75% luminal narrowing (Fig. 1).

Fluid deficit was corrected by glucose-normal saline. His blood pressure dropped from 205/150 mmHg to 160/100 mmHg. Intravenous hydralazine and oral slow-release nifedipine 20 mg were started. Systolic blood pressure gradually decreased to 110–125 mmHg after adding oral methyldopa, carvedilol and captopril. As BP improved, serum sodium levels partially normalized and polyuria gradually resolved. Hypokalemia was corrected intravenously.

Renal Tc-99 m DTPA scan revealed that the left kidney accounted for only 14% of total renal function. A left nephrectomy was performed, taking into careful consideration the technical difficulties of angioplasty or artery reconstruction because of the very close proximity of the stenotic segment to the aortic ostium. Within a week, the patient showed complete resolution of polyuria, polydipsia, hypertension, and electrolyte disturbances, and all antihypertensive medications were discontinued.

At three-month follow-up, the patient remained seizure-free. His weight had increased to 16 kg, and blood pressure was normal without the need for antihypertensive medications.

## Lessons for the clinical nephrologist

Hyponatremic hypertensive syndrome was first reported in adults, and is a rare condition in children. It is typically characterized by hypertension and hyponatremia and can be caused by a number of disorders including kidney failure, malignant hypertension, thiazide use, renin-secreting tumors and renal ischemia [1]. Hyponatremic hypertensive syndrome caused by renal ischemia is rare [2]. The commonest etiology in children is unilateral renal artery stenosis. Early diagnosis and timely treatment are crucial to avoid complications such as posterior reversible encephalopathy syndrome, which may arise due to uncontrolled hypertension combined with cerebral edema from hyponatremia [3]. Hyponatremic hypertensive syndrome usually presents with neurological symptoms such as headache, confusion, and seizures, along with weight loss, polydipsia, and polyuria. Laboratory findings include hyponatremia, hyperreninemia, hypokalemia, hyperaldosteronism, metabolic alkalosis, and increased urinary sodium and protein excretion [1].

Renovascular hypertension should be considered in children with suspected secondary hypertension, particularly when associated with elevated plasma renin, hypokalemia, or severe hypertension requiring more than two antihypertensive agents to be controlled [4]. A number of causes of renovascular disease are shown in Table 1S (supplemental material).

This is a case of 5-year-old male who presented with seizures and severe hypertension. Coarctation of the aorta was unlikely due to the absence of blood pressure discrepancies between the upper and lower limbs, which was further confirmed by echocardiography. There were no signs of systemic or renal parenchymal disease. The presentation with hypertension, hyponatremia, hypokalemia, and polyuria, strongly suggested hyponatremic hypertensive syndrome. Brain MRI findings were consistent with posterior reversible encephalopathy syndrome.

**Fig. 2:**
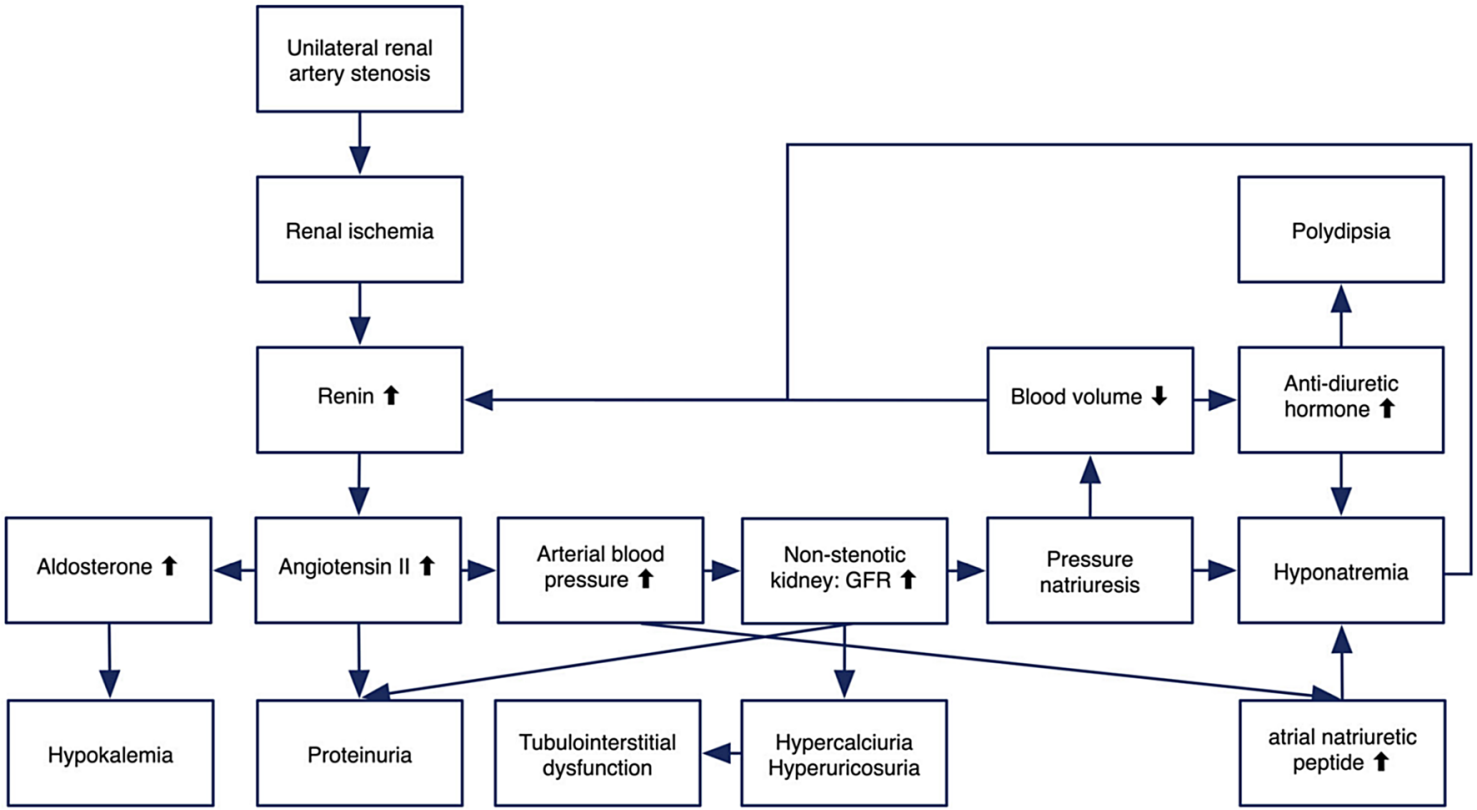
Possible mechanism of hyponatremic-hypertensive syndrome [3]

The main pathogenesis of hyponatremic hypertensive syndrome is renal ischemia, as shown in Fig. 2 [3]. Renal ischemia leads to increased renin secretion, which elevates angiotensin II levels. This, in turn, raises blood pressure through direct vasoconstriction and secondary hyperaldosteronism. A rapid rise in arterial pressure can cause glomerular hyperfiltration and pressure natriuresis in the non-stenotic kidney, causing volume depletion and hyponatremia, which may result in further renin release from the ischemic kidney [5]. Hypokalemia, resulting from hyperaldosteronism, exacerbates renin secretion, thus intensifying the vicious circle. Additionally, hyponatremia can be further exacerbated by stimulation of thirst and antidiuretic hormone release, triggered by both high angiotensin II levels and volume depletion. Metabolic alkalosis is also a consequence of renin–angiotensin–aldosterone system activation [3]. Glomerular hyperfiltration in the contralateral healthy kidney may lead to tubulointerstitial damage by hypercalciuria and hyperuricosuria. Proteinuria in hyponatremic hypertensive syndrome can result from glomerular hyperfiltration, the proteinuric effects of angiotensin II, or tubulointerstitial injury from hypercalciuria and hyperuricosuria [3].

Ding et al. [3] reviewed 15 pediatric cases of hyponatremic hypertensive syndrome secondary to unilateral renal artery stenosis, reporting a mean age of onset of 4.03 ± 3.38 years and a male predominance (11 out of 15), consistent with the age and gender of our patient. The most common clinical features were hypertension, polydipsia, and polyuria (14/15), followed by hyponatremic seizures (7/15). Similarly, Agarwal et al. [2] described 32 adult cases, with predominant symptoms including headache, altered consciousness, or confusion (24/32), and features such as weakness, weight loss, thirst, and polyuria (15/32). Papilledema or retinal hemorrhage was seen in 7 patients. Our patient had comparable findings, with hypertensive retinopathy observed on fundus examination of the left eye.

An important differential diagnosis for this presentation includes causes of hypokalemic hypertension. Liddle syndrome, resulting from mutations in the epithelial sodium channel, leads to aldosterone-independent sodium reabsorption in the distal nephron. It typically presents with hypertension, polyuria, hypokalemia, and metabolic alkalosis, along with suppressed plasma renin activity and low aldosterone levels, often accompanied by hypernatremia. Similarly, apparent mineralocorticoid excess, an autosomal recessive disorder caused by mutations in the *HSD11B2* gene encoding 11β-hydroxysteroid dehydrogenase type 2, presents with juvenile-onset severe hypertension, low renin and aldosterone, hypokalemia, and metabolic alkalosis. Familial hyperaldosteronism also leads to hypokalemic hypertension and metabolic alkalosis, typically associated with inappropriately elevated aldosterone levels and hypernatremia.

The management of hyponatremic hypertensive syndrome secondary to renal artery stenosis involves correction of volume depletion and hypokalemia, blood pressure control, and definitive treatment of the underlying renal artery stenosis. Restoring intravascular volume is a priority to improve systemic perfusion and minimize further renal ischemic injury [6]. Hypertension can be controlled by administering intravenous calcium channel blockers, while diuretics are avoided to prevent exacerbation of fluid and sodium loss and further stimulation of the renin–angiotensin–aldosterone system [7]. Angiotensin-converting enzyme inhibitors and angiotensin II receptor blockers should be introduced in patients with hyponatremic hypertensive syndrome to block the over-activation of the renin–angiotensin–aldosterone system. However, they are generally contraindicated as first line antihypertensives, particularly in bilateral disease or in patients with a solitary kidney [4]. Surgical correction of renal artery stenosis can be achieved by renal angioplasty, renal artery reconstruction or unilateral nephrectomy, with the latter considered when the affected kidney contributes less than 10% to total kidney function or if percutaneous angioplasty fails [8]. In our case, the patient was initially stabilized with intravenous hydralazine, beta-blockers, captopril and calcium channel blockers, followed by successful management with left nephrectomy.

Delayed recognition and inadequate treatment of hyponatremic hypertensive syndrome can result in irreversible renal ischemia, end-organ damage, such as left ventricular hypertrophy, and neurological injury from hypertensive or hyponatremic encephalopathy [6]. The prognosis for posterior reversible encephalopathy syndrome associated with renal disease is generally favorable, with most patients recovering without lasting neurological deficits [9].

In conclusion, this rare case of unilateral renal artery stenosis in a young child emphasizes the importance of early recognition and appropriate management. This report contributes to the limited pediatric literature on hyponatremic hypertensive syndrome and advocates for heightened awareness among pediatricians managing hypertension.

## Supplementary Information

Below is the link to the electronic supplementary material.

Supplementary file1 (DOCX 282 KB)

## Data Availability

The data underlying this article will be shared on reasonable request to the corresponding author.
