# Supplementary material for: Lessons for the clinical nephrologist: unilateral renal artery stenosis presenting with hyponatremic hypertensive syndrome and posterior reversible encephalopathy syndrome in a child
Source: J Nephrol. 2025 Jun 10;38(8):2453–7. doi: 10.1007/s40620-025-02320-7 (PMC12630290; doi:10.1007/s40620-025-02320-7)
Supplement: Supplementary file 1 — Supplementary file1 (DOCX 282 KB) [file 40620_2025_2320_MOESM1_ESM.docx]

**Fig. S1** MRI brain showing small area of demyelination at left parieto-occipital region consistent with PRES.


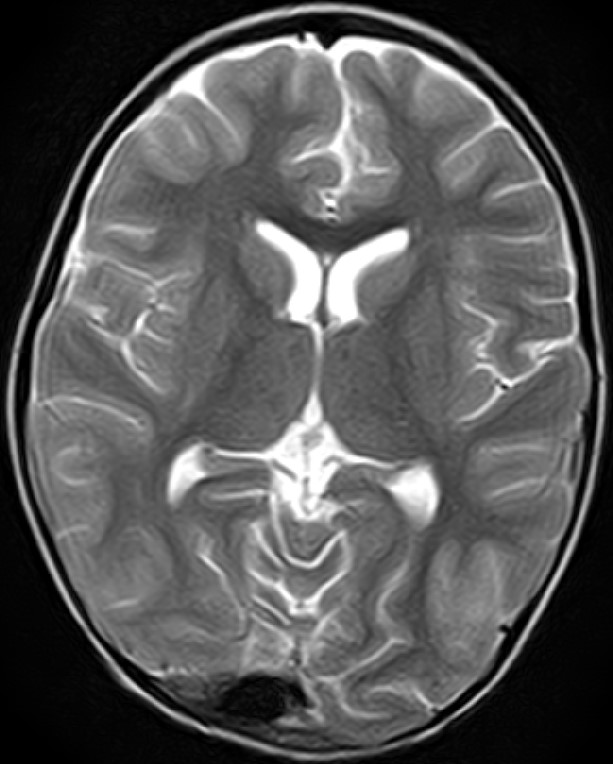

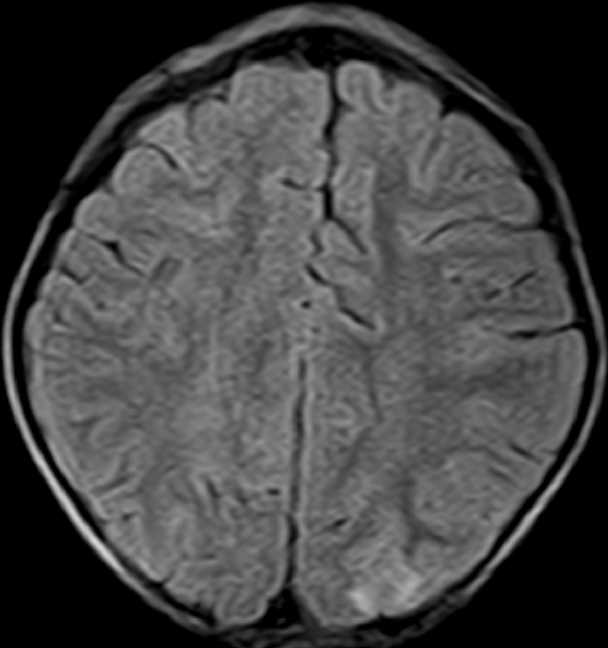


**Table S1** Causes of renovascular hypertension in the pediatric population [1]

| **Categories** | **Specific etiologies** |
| --- | --- |
| Anatomical | Fibromuscular dysplasia, extrinsic compression |
| Vasculitis | Kawasaki disease, polyarteritis nodosa, Takayasu’s disease |
| Syndromes | Neurofibromatosis 1, tuberous sclerosis, Marfan’s syndrome, William’s syndrome |
| Localized tissue damage | Trauma, radiation, umbilical artery catheterization |
| Congenital | Congenital rubella |

1.     Parikh P, Duhame D, Monahan L, Woeoniecki R. Renal artery stenosis precipitates hyponatremic hypertensive syndrome and posterior reversible leucoencephalopathy. Front Pediatr. 2015;3:40.
